# Epidemiology of Respiratory Infections during the COVID-19 Pandemic

**DOI:** 10.3390/v15051160

**Published:** 2023-05-13

**Authors:** Nicola Principi, Giovanni Autore, Greta Ramundo, Susanna Esposito

**Affiliations:** 1 Università degli Studi di Milano, 20122 Milan, Italy; nicola.principi@unimi.it; 2 Pediatric Clinic, Pietro Barilla Children’s Hospital, Department of Medicine and Surgery, University Hospital of Parma, 43126 Parma, Italy; giovanniautore@gmail.com (G.A.);

**Keywords:** COVID-19, group A *Streptococcus*, *Streptococcus pneumoniae*, influenza, respiratory infection, RSV, SARS-CoV-2

## Abstract

To face the COVID-19 outbreak, a wide range of non-pharmaceutical interventions (NPIs) aimed at limiting the spread of the virus in communities, such as mask-wearing, hand hygiene, social distancing, travel restrictions, and school closures, were introduced in most countries. Thereafter, a significant reduction of new asymptomatic and symptomatic COVID-19 cases occurred, although there were differences between countries according to the type and duration of the NPIs. In addition, the COVID-19 pandemic has been accompanied by significant variations in the global incidence of diseases due to the most common non-SARS-CoV-2 respiratory viruses and some bacteria. In this narrative review, the epidemiology of the most common non-SARS-CoV-2 respiratory infections during the COVID-19 pandemic is detailed. Moreover, factors that could have had a role in modifying the traditional circulation of respiratory pathogens are discussed. A literature analysis shows that NPIs were the most important cause of the general reduction in the incidence of influenza and respiratory syncytial virus infection in the first year of the pandemic, although the different sensitivity of each virus to NPIs, the type and duration of measures used, as well as the interference among viruses may have played a role in modulating viral circulation. Reasons for the increase in the incidences of *Streptococcus pneumoniae* and group A *Streptococcus* infections seem strictly linked to immunity debt and the role played by NPIs in reducing viral infections and limiting bacterial superimposed infections. These results highlight the importance of NPIs during pandemics, the need to monitor the circulation of infectious agents that cause diseases similar to those caused by pandemic agents, and the need to make efforts to improve coverage with available vaccines.

## 1. Introduction

After its first detection in China in December 2019, severe acute respiratory syndrome coronavirus 2 (SARS-CoV-2) reached all countries worldwide within a few weeks, leading the World Health Organization (WHO) to declare the COVID-19 outbreak a global pandemic on 11 March 2020 [1]. In the first months, due to the free circulation of the wild SARS-CoV-2 strain, the pandemic resulted in a number of devastating medical, social, and economic effects [2]. To face these problems, a wide range of non-pharmaceutical interventions (NPIs) aimed at limiting the spread of the virus in communities, such as mask-wearing, hand hygiene, social distancing, travel restrictions, and school closures, were introduced in most countries [3,4,5,6,7,8]. Thereafter, a significant reduction of new asymptomatic and symptomatic COVID-19 cases occurred, although there were differences between countries according to the type and duration of NPIs [9,10]. However, starting from the last part of 2020, previously implemented NPIs were progressively reduced and SARS-CoV-2 variants with increased transmissibility and infectivity emerged, radically changing the course of the pandemic [11]. The Delta and Omicron variants were the most important in this regard. The Delta variant replaced previous circulating strains, becoming the only cause of COVID-19 in most countries in the second part of 2021. Moreover, starting from the end of 2021, the Omicron variant was detected and it, with its subvariants, is presently the most common SARS-CoV-2 circulating strain [12]. The emergence of dangerous variants has been systematically associated with new COVID-19 waves and increases in COVID-19-related problems [13].

Fortunately, several factors have significantly mitigated the epidemiological and clinical relevance of new waves. People who have already been infected with SARS-CoV-2 have a lower risk of being newly infected, although the immune response evoked by the previously circulating strains could be inadequate at preventing new variant infections [14]. Several sensitive and specific point-of-care tests were developed and introduced in clinical practice [15], allowing tracing of infected people, isolation, and reduction of virus circulation. Finally, several safe and effective vaccines [16,17], monoclonal antibodies [18,19], and antivirals [20,21] were developed and rapidly authorized for emergency use by health authorities. Although the final effectiveness of these preparations, particularly against the most recent variants, may be lower than desired, as some of the variants escape from protection induced by vaccination and monoclonal antibodies [22,23], they have had a dramatic positive effect. In the USA, it has been calculated that two doses of mRNA vaccines administered to subjects ≥18 years of age have reduced the overall attack rate of SARS-CoV-2 infection from 9% to 4.6% for over 300 days, decreasing non-ICU hospitalizations, ICU hospitalizations, and deaths by 63.5%, 65.6%, and 69.3%, respectively [24].

The COVID-19 pandemic has been accompanied by significant variations in the global incidence of diseases due to the most common non-SARS-CoV-2 respiratory viruses [25,26] and some bacteria [27,28]. The incidence of respiratory infections was initially reduced, although with differences between infecting agents. As the pandemic progressed, the circulation of the various non-SARS-CoV-2 infectious agents varied significantly, in most cases independently of COVID-19 waves. A rapid return to traditional levels was evidenced in some cases. In other cases, low circulation persisted for several months and a return to pre-pandemic levels was reached only after three pandemic years [25,26,27,28]. Several factors could explain these findings, including the implementation of the various NPIs and the duration of their use. Knowledge of the impact of the COVID-19 pandemic on the epidemiology of respiratory infections due to non-SARS-CoV-2 agents and the importance of factors leading to infectious agent epidemiology variations can be very useful to understand infectious agent interrelationships, the best measures to limit their spread, and the best moment to plan preventive measure use, when available.

In this narrative review, the epidemiology of the most common non-SARS-CoV-2 respiratory infections during the COVID-19 pandemic is detailed. Moreover, factors that could have played a role in modifying the traditional circulation of respiratory pathogens are discussed. An in-depth review of the medical literature was performed. The MEDLINE/PubMed database was searched from 2014 to 31 March 2023 to collect the literature. The search included randomized placebo-controlled trials, controlled clinical trials, double-blind, randomized controlled studies, and systematic reviews of the last five years. The following combinations of keywords were used: “COVID-19” AND “respiratory viruses” AND/OR “influenza” AND/OR “RSV” AND/OR “influenza” AND/OR “enveloped viruses” AND/OR “non-enveloped viruses” AND/OR “*Streptococcus pneumoniae*” AND/OR “*Streptococcus pyogenes*”. We also performed a manual search of the reference lists of the obtained studies. The search was limited to English-language journals and full papers only.

## 2. Circulation of Non-SARS-CoV-2 Respiratory Viruses during the COVID-19 Pandemic

Significant variations in the epidemiology of all of the most common respiratory viruses were evidenced during the years of the COVID-19 pandemic. However, fluctuations varied significantly among viruses, particularly between enveloped and non-enveloped viruses.

### 2.1. Epidemiology of Enveloped Viruses during the COVID-19 Pandemic

#### 2.1.1. Influenza Viruses

Reduced activity of influenza viruses was reported worldwide as early as a couple of weeks after NPI implementation, remaining significantly below the values recorded in the years preceding the pandemic up to the entire 2020–2021 season [29]. The activity of influenza viruses slowly increased in the following months, but the number of influenza diagnoses did not reach pre-pandemic values even in the 2021–2022 influenza season, despite the fact that NPIs had been significantly reduced or eliminated in most countries. Variations occurred all over the word as they were detected in several countries in both the northern hemisphere and, compatibly with the different temporal distribution of the influenza season during the year, the southern hemisphere. Data collected in the USA are a good example in this regard. In this country, compared to the period 29 September 2019–29 February 2020, in the period 1 March–16 May 2020 the number of respiratory specimens submitted to clinical laboratories for virus identification and the number of those positive for influenza declined by 61% and 98% respectively. Moreover, summer circulation of influenza viruses in 2020 was among the lowest in the country’s history, as samples positive for influenza were only 0.20%, compared to 2.35% in 2019, 1.04% in 2018, and 2.36% in 2017 [30]. Influenza activity was also significantly reduced in the 2020–2021 season, when the positivity of tested laboratory samples remained limited to only 0.20% [31]. The circulation of influenza viruses increased slowly during the following months, but even in the 2021–2022 influenza season it remained significantly lower than that recorded in pre-pandemic years. Clinical laboratories and public health laboratories reported 4.5% and 2.8% positive tests, respectively, compared to values of about 30% in pre-pandemic years [32]. Interestingly, the 2021–2022 influenza season occurred in two waves, with the second wave starting in March 2022 and remaining elevated until mid-June 2022, as if, with the attenuation of the pandemic, a lengthening of the traditional length of the influenza season had occurred. Regarding the 2022–2023 influenza season, it is expected that influenza activity could significantly increase, reaching values quite similar or even higher than those seen in pre-pandemic years. Data reported to the CDC indicate that in the USA, in the period October 2002–4 March 2023, 12.8% of tested respiratory samples resulted positive, with a peak value of more than 25% in December, 2022 [33].

Variations in influenza virus circulation were accompanied by similar variations in the number of emergency department (ED) visits, hospital admissions, and deaths associated with influenza infection. In the USA, during late 2019 and early 2020, before COVID-19 pandemic declarations, all the clinical markers of influenza activity had values like those reported in previous years [34]. With the introduction of NPIs, the number of ED visits and hospitalizations for influenza declined to extremely low levels. During June 2020–March 2021, ED visits for influenza accounted for less than 0.1% of all visits compared to 5% reported in February 2019 [35]. Hospitalization rates were only 0.8 per 100,000 population (and deaths were not calculated due to the low numbers of reported hospitalizations) [36]. During the 2021–2022 influenza season, emergency department visits, hospitalizations, and deaths also increased with the increase in influenza virus circulation, although they remained largely below pre-pandemic levels. Hospitalizations were 17.1 per 100,000 population, compared to 62.0 per 100,000 and 102.9 per 100,000 reported in the 2016–2017 and 2019–2020 influenza seasons, respectively. Deaths were 9000 compared to 38,000 and 25,000 [36]. For the 2022–2023 season, CDC estimates predict 12–24 million medical visits, 290,000–620,000 hospitalizations, and 18,000–55,000 deaths, values not substantially different from those reported before the pandemic [37].

Regarding the impact of the COVID-19 pandemic on the circulation of influenza virus subtypes, several studies have shown that, when influenza activity started to grow again after the initial sharp reduction, only very few cases of the B/Yamagata lineage were reported, and no virus of this subtype was isolated and sequenced [38]. This suggests that this influenza lineage may have become extinct.

#### 2.1.2. Respiratory Syncytial Virus (RSV)

As for influenza, during the first winter period after the COVID-19 pandemic declaration the incidence of RSV infection was found to be significantly lower than in the pre-pandemic periods in all the countries in which reliable data could be collected, regardless of whether they were located in the northern or southern hemisphere. A study carried out in England that enrolled children < 5 years of age showed that, compared to pre-pandemic corresponding periods, during winter 2020–2021 RSV laboratory-confirmed cases, hospital admissions, and emergency department attendances were reduced by 99.6%, 80.8%, and 85.3%, respectively [39]. In Japan, no RSV outbreak was reported in 2020 [40]. In Australia, RSV detection for the 2020 winter season (from April to June) had a 93.4% reduction, concomitantly with 85.9% and 89.1% reductions of bronchiolitis cases requiring hospitalization or admission to the ICU, respectively [41]. However, in 2021, early epidemics of RSV infection were evidenced in several countries, with virus detection rates in some cases exceeding the seasonal peak of pre-pandemic years. In England, compared to predicted cases, laboratory-confirmed cases, the number of emergency department attendances for acute bronchitis or bronchiolitis, and the total number of hospital admissions for RSV-attributable respiratory disease increased by more than 100%, 84.9%, and 10.7%, respectively [39]. In the USA, where the total number of RSV infections had remained lower than expected until early 2021, a sharp increase was shown in summer 2021, largely before the traditional RSV season [42]. Substantial similar rebounds were also reported in Japan [40] and Australia [41]. In Italy, a study carried out in four pediatric hospitals showed an early RSV circulation beginning in early autumn 2021 and lasting until early winter, when an abrupt decrease, concurrent with a local sudden increase in the number of SARS-CoV-2 pediatric infections, occurred [43]. Early RSV epidemics were also reported in 2022 in many countries, and the number of infections and hospital admissions were substantially similar or even greater than those during the typical winter peaks. In the USA, the CDC reported that on 4 November 2022 the incidence of RSV had already been increasing in most US health regions, with values quite similar to those usually observed during the virus’s seasonal peak month of December or January [44]. In Denmark, where in 2021 RSV presented as a highly unusual out-of-season epidemic, a similar early increase of RSV cases was detected from mid-August [45]. The epidemic reached its peak in November, when the first RSV cases are detected in traditional RSV seasons.

Together with fluctuations in the circulation of RSV, COVID-19 was associated with changes in RSV subtype predominance. In Australia, where before the COVID-19 pandemic RSV-A and RSV-B co-circulated with similar prevalence, after the COVID-19 pandemic onset and the introduction of NPIs there was evidence of the absolute predominance of RSV-A [46].

### 2.2. Epidemiology of Non-Enveloped Viruses during the COVID-19 Pandemic

#### Rhinovirus/Enterovirus, Adenovirus, and Bocavirus

During the COVID-19 pandemic, the circulation of non-enveloped viruses (i.e., rhinovirus/enterovirus, human bocavirus, and human adenovirus) was different from that of enveloped viruses worldwide. Differences in epidemiology were particularly evident for rhinovirus/enterovirus, the viruses of this group that play the major role as causes of disease and are more frequently studied [47,48,49,50,51,52,53,54]. At the onset of the pandemic and after the introduction of NPIs, detection of non-enveloped viruses in patients with respiratory infection declined, as did that of all enveloped viruses. However, after only a few months, despite the persistence or only slight attenuation of NPIs, detection of non-enveloped viruses progressively increased, returning to pre-pandemic or even higher levels in most countries [47,48,49,50,51,52,53,54]. In the USA, a multicenter study enrolling 38,198 pediatric patients requiring medical care for fever and/or respiratory symptoms compared the frequency of rhinovirus/enterovirus infection between December 2016 and February 2021 [55]. The study showed that in the first months of the pandemic, from April to September 2020, rhinovirus and/or enterovirus detection occurred at lower levels than in pre-pandemic years (odds ratio [ORs]: from 0.08 to 0.76 in emergency departments and from 0.04 to 0.71 in hospitals). However, unlike some other viruses, rhinoviruses and/or enteroviruses soon returned to pre-pandemic levels. From October 2020 to February 2021, detection of these agents was found more commonly than in pre-pandemic months (ORs: from 1.47 to 3.01 in EDs and from 1.36 to 2.44 in hospitals). Moreover, although in all periods most cases occurred in children younger than 5 years, the mean age of infected children was slightly higher [55].

Similar findings were reported in studies evaluating adenovirus circulation. The European Non-Polio Enterovirus Network signaled that, after its disappearance in the first COVID-19 pandemic year, enterovirus D68 (EV-D68) reappeared in 2021 [56]. A greater number of infections than were diagnosed in the years before were identified between 31 July and 14 October 2021 in several European countries. In the USA, after an extended period of low EV-D68 circulation, the incidence of EV-D68 infections significantly increased in the period July-September 2022, leading to fears of an increase in cases of acute flaccid myelitis and severe respiratory illness potentially associated with this infectious agent [57].

Sustained non-enveloped virus circulation was associated with relevant clinical consequences. Rhinovirus/enterovirus infections are frequently associated with the development of wheezing and the exacerbation of asthma [58,59,60]. In the USA, visits for acute respiratory infection and asthma/reactive airway disease from week 1 of 2018 through week 37 of 2022 were analyzed [57]. They show that, in summer 2022, concomitantly with the increase in the number of rhinovirus/enterovirus infections, a relevant increase in the number of emergency department visits for these diseases occurred. In particular, visits for asthma/reactive air diseases were about three times more common than in 2020, when circulation of rhinovirus/enterovirus was very low [57].

## 3. Reasons for Variations in Epidemiology of Non-SARS-CoV-2 Viruses during the COVID-19 Pandemic

The dramatic reduction in the circulation of all the non-SARS-CoV-2 respiratory pathogens seen worldwide in the first months after the onset of the COVID-19 pandemic should mainly be attributed to the introduction of NPIs. No new preventive pharmacological measures against the infections caused by these pathogens had been introduced and influenza vaccination coverage, albeit with differences among countries, generally aligned with local vaccination history or was only slightly higher than in the pre-pandemic era [61,62]. However, defining which were the most effective measures and how much they affected the spread of the pathogens, together with the containment of COVID-19 cases, remains very difficult, if not impossible [63]. The planning and type of NPIs were very different from country to country. Moreover, when the implementation of NPIs was similar in type and month of intervention, definitive conclusions could not be drawn, mainly due to the complexity of efficacy assessments [64,65]. On the other hand, similar findings had already been reported in the past when the role of NPIs in controlling influenza epidemics was studied [66]. Several factors besides the type of NPI may play a role in influencing infectious agent circulation, making the evaluation of NPI impact very difficult. Among these, the most important are the pathogen’s characteristics, the type of involved population, and the location and time of NPI implementation [67]. Transmissibility and mode of transmission are specific to each wild virus and may vary among virus variants and subvariants [68]. Development of an infection is strictly dependent on the susceptibility to infection of the healthy population and the mode of contact between the infected patient and the exposed subject. Personal protective measures can be effective in reducing individual risk but have a poor capacity to protect society if they are not fully and continuously used. Environmental measures, social distancing, and movement restrictions can significantly reduce virus circulation, but they can be implemented only for a short time as they can cause severe social and economic problems. The timing of NPI introduction can be critical. Where interventions are enacted early, the spread of all respiratory viruses are more effectively controlled, and emergency department visits, hospitalizations, and deaths for respiratory diseases are markedly reduced. The reverse occurs when implementation is late [69].

Although the true role of NPIs in reducing the circulation of respiratory non-SARS-CoV-2 infectious agents in the first phase of the COVID-19 pandemic cannot be precisely defined, it is indisputable that these measures were effective globally. A decline in respiratory disease rates occurred immediately after NPIs were implemented worldwide. Moreover, many viruses resumed substantial circulation as soon as NPIs were reduced. However, the evidence that, at least in some cases, non-SARS-CoV-2 virus epidemiology remained partly independent from NPIs and resumed traditional characteristics in different time periods regardless of COVID-19 waves and NPI reintroduction suggests that other factors may have played a relevant role in conditioning infectious agent circulation during the COVID-19 pandemic. Differences in transmissibility between viruses may explain differences in epidemiology. Compared to enveloped viruses, non-enveloped viruses are generally more virulent, are shed from infected individuals for a significantly longer time, can survive in the gastrointestinal tract, and are more resistant to extreme pH, heat, dryness, and simple disinfectants [70]. All these factors favor the persistence of these viruses on surfaces and extended shedding among infected subjects, explaining why the circulation of rhinovirus/enterovirus, human bocavirus, and human adenovirus was only marginally influenced by NPIs and returned to traditional levels even when these measures were still in place or were marginally lifted [71,72]. Enveloped viruses, which have the opposite characteristics, were significantly influenced by NPIs. The circulation of these viruses was significantly reduced or totally abolished immediately after these measures were implemented. Later, when NPIs were partially or totally removed, the circulation increased, although the speed with which it returned to pre-pandemic levels differed significantly. Influenza virus activity increased slowly, whereas RSV activity had a sharp increase, leading to early and very strong epidemics already in the second year of the pandemic. Also in this case, the greater sensitivity of RSV to NPIs compared to the influenza viruses may explain the total disappearance of RSV in the first COVID-19 year. However, with the lifting of NPIs, RSV began to circulate again, causing sudden and great interseasonal epidemics mainly involving children aged <4 years and old adults [73]. This is because in the absence of viral exposure no specific protective immune response in women of childbearing age and infants born in 2020 was established. Moreover, as immunity due to RSV infection is relatively short-lived and maintaining protection requires repeated viral exposure, a great number of children born in the years before the onset of the pandemic and old people remained susceptible and were infected as soon as the virus started to circulate again [74,75].

Variations in non-SARS-CoV-2 virus epidemiology may also depend on antagonistic competition between SARS-CoV-2 and other respiratory viruses or between two or more of these infectious agents. It has been shown that subjects with influenza have a low risk of COVID-19 [76] and that a similar correlation can be demonstrated for COVID-19 and rhinoviruses and for influenza and rhinoviruses [77]. However, it has been shown that the degree of interference is virus-specific and that influenza A viruses are more effective than RSV in blocking SARS-CoV-2 [78].

Finally, as already reported for RSV, it has been suggested that the susceptibility of the host to a given virus may condition circulation of the virus itself and contribute to the modification of the circulation of one or more other respiratory agents. For example, it has been supposed that the large use of the influenza vaccine during the influenza season 2020–2021 may have favored not only the incidence of influenza cases but may have also played a role in the circulation of those viruses that can benefit from the disappearance of influenza viruses [79]. However, reasons for variations in the circulation of respiratory virus subtypes such as those evidenced for RSV and influenza viruses are difficult to explain. As far as the disappearance of the influenza B/Yamagata subtype is concerned, it has been supposed that, as this virus has a shorter transmission chain, it was more sensitive to NPIs [80].

## 4. SARS-CoV-2 Circulation and Incidence of Bacterial Infections

### 4.1. Streptococcus pneumoniae Infections

Several studies have shown that in 2020, during the first months of the COVID-19 pandemic, the incidence of *S. pneumoniae* invasive diseases (IPDs), including bacteremic pneumonia, was significantly reduced, particularly in children < 5 years of age [81,82,83,84,85,86,87]. This finding was generally attributed to the impact of NPIs, although it was not definitively clarified whether the greater role was played by the reduction of interpersonal transmission of the pathogen or by the suppression of the activity of several seasonal respiratory viruses. Infections by influenza viruses, RSV, and hMPV favor pneumococcal colonization and are frequently complicated by *S. pneumoniae* superinfection [24,27,88,89,90,91]. Results of the study by Danino et al. significantly contribute to solving the problem [92]. These authors studied monthly rates of supposed or documented bacterial and viral CAPs, nasopharyngeal pneumococcal carriage, nasopharyngeal respiratory virus evidence, and nationwide IPDs in children < 5 years in the first years of the COVID-19 pandemic and in the 4 years before. A comparison showed that the incidences of both pneumococcal CAP and viral CAP were strongly reduced concomitantly with a full suppression of RSV, influenza viruses, and hMPV circulation. On the contrary, carriage prevalence was only marginally reduced, without differences in density of carriage and serotype distribution, showing that reduction of pneumococcal IPDs during the first phase of the COVID-19 pandemic were mainly due to the suppression of viral infections rather than increased bacterial transmission.

However, the incidence of IPDs totally changed during the second year of the pandemic, when the NPIs imposed by the health authorities were partially or totally removed. A study conducted in England showed that IPD incidence in children < 15 years of age from July to December 2021 was about three times higher than that observed in the same period of 2020 (1.96/100,000 children vs. 0.7/100,000 children) and higher than that calculated in the pre-pandemic years of 2017–2019 (1.43/100,000 children) [93]. The association of the reemergence of IPDs with the documented early and severe increase in RSV cases in 2021 among children seems to confirm the strict relationship between the circulation of some respiratory viruses and IPD development [94]. However, in England, a role in IPD increase may have been played by a substantial reduction of pneumococcal vaccination coverage [95]. Similar findings were reported in a study carried out in Germany in which it was found that among patients of any age, including children 0–4 years old, the incidence of IPDs progressively increased starting from spring 2021, reaching values quite similar or above those evidenced in the pre-pandemic years of 2015–2019 during the summer [96]. As in England, in Germany the reemergence of IPDs can be related to the dynamic of viral infections favoring *S. pneumoniae* colonization and disease. In that country, the incidence of influenza-like illness, which was reduced during the 2020–2021 winter season, returned to traditionally reported values in spring 2021, when IPDs reappeared. Moreover, RSV circulated well in advance, resulting in a high number of hospital admissions already during the summer and coinciding with the return of IPD incidence to its highest values [96].

### 4.2. Group A Streptococcus Infections (GAS)

As for *S. pneumoniae*, during the first phase of the COVID-19 pandemic the incidence of GAS diseases declined as a consequence of NPI introductions [97]. However, in several countries, a relevant increase of GAS infections was documented during the second half of 2022, reaching values even higher than those reported in pre-pandemic years. In England, the number of notifications of scarlet fever and invasive GAS infections was several times higher than in previous years in all age groups, with the greatest increase in children. Compared to the previous five years, during the 2022–2023 season the incidence of invasive GAS infection was 2.5 vs. 0.2–1.3/100,000 in children < 1 year old, 4.1 vs. 0.1–1.3/100,000 in children 1–4 years old, and 1.8 vs. 0.1–0.6/100,000 in those aged 5–9 years. Moreover, GAS infections were associated with a greater risk of death than they had been previously [97]. Similar findings were reported in several European countries such as France, Ireland, the Netherlands, and Sweden, with children under 10 years of age the most affected [98]. Finally, in the USA, increases in invasive GAS infections were detected in several children’s hospitals, leading the Centers for Disease Control and Prevention (CDC) to declare that invasive GAS infections in children have returned to levels similar to those seen in pre-pandemic years [99].

Different factors may have contributed to increasing GAS infection rates. The emergence of a new GAS strain was excluded as bacterial types detected in patients recently diagnosed were substantially similar to those detected before the pandemic declaration, and no unusual strain was detected [100]. In England, the same toxigenic GAS emerged in 2014 and was later detected in cases diagnosed in 2022, prior to the COVID-19 pandemic [97]. A role may have been played by the so-called immunity debt, i.e., the lack in many children of any experience with GAS due to NPIs. Decreased exposure to GAS would have prevented the development of any form of immunity against GAS, thus favoring colonization and development of diseases due to this pathogen [101]. However, it cannot be excluded that the increase in viral respiratory infections could have favored, as already reported for *S. pneumoniae*, a superimposed GAS infection. Children with scarlet fever or invasive GAS infection frequently have a concomitant viral respiratory infection. In the English study, 25.8% of <15-year-old children with invasive GAS had a documented co-infection [97]. RSV was detected in 12.4% of the cases, hMPV in 11.3%, and rhinovirus in 7.2% [34]. Co-infection was demonstrated in 5 out of 10 deceased children. As increases in GAS infections occurred contextually with increases in pediatric RSV and rhinovirus infections, it can be assumed that the two events were closely related and that the viral infection has, in most of the cases, favored the bacterial superimposed infection.

## 5. Conclusions

During the COVID-19 pandemic, circulation of SARS-CoV-2 was associated with significant modifications of the circulation of non-SARS-CoV-2 respiratory viruses and some bacteria. The reasons for the increase in the incidences of *S. pneumoniae* and GAS infections are easier to explain, as they seem strictly linked to the role played by NPIs in reducing viral infections and limiting bacterial superimposed infections. Moreover, at least in some cases, poor exposure to *S. pneumoniae* and GAS due to NPI implementation may have led to a significant immunity debt in relevant population groups, favoring infection development. Much more difficult to explain are the reasons for non-SARS-CoV-2 virus fluctuations. It seems indisputable that NPIs were the most important cause of the general reduction in the incidence of infections in the first year of the pandemic, although the different sensitivities of each virus to NPIs and the type and duration of measures used may have played a role in modulating virus circulation. What happened later, in the second and third years of the pandemic, is more difficult to understand. These later developments have been the consequence of the intersection of various factors, each capable of modifying the traditional epidemiology of individual viruses with results in which the causative role of any single component is not precisely defined. Effects of NPIs that were reduced, abolished, or transiently reactivated according to the emergence of viral variants and the development of new COVID-19 waves have been associated with the effects of the changed immune protection of the population towards some viruses because of vaccinations in the case of influenza and SARS-CoV-2 and immunity debt in the case of RSV. Furthermore, a role may have been played by the interference among viruses. Despite these limitations, findings of the studies discussed in this paper have important practical implications. First of all, they highlight the importance of NPIs during pandemics; however, because the importance of the various measures and their simultaneous implementation and duration were not precisely defined, they should stimulate institutions to evaluate and improve best practices for facing potential future pandemics. Moreover, the results indicate that monitoring the circulation of infectious agents that cause diseases quite similar to those caused by the pandemic agent is mandatory in order to quickly understand which unexpected diseases may emerge. Finally, due to the risk of viral interference or bacterial superimposed infections, any effort to improve coverage of available vaccines should be made.

## Data Availability

Not applicable.
